# Anemia and Cost-Effectiveness of Complete Blood Count Testing Among Pregnant Women at King Abdulaziz University Hospital: A Single Tertiary Center Experience

**DOI:** 10.7759/cureus.10493

**Published:** 2020-09-16

**Authors:** Anas M Fallatah, Anas E Bifari, Hisham Z Alshehri, Sahal M Wali, Saleh A Alghamdi, Sultan A Almusallam, Wid S Al-Abbadi, Samera Albasri

**Affiliations:** 1 College of Medicine, King Abdulaziz University, Jeddah, SAU; 2 Department of Radiology, King Abdulaziz University Hospital, Jeddah, SAU; 3 Radiology, King Abdulaziz University, Jeddah, SAU; 4 Obstetrics and Gynecology, King Abdulaziz University Hospital, Jeddah, SAU

**Keywords:** anemia, maternal, neonatal, pregnancy outcomes, cbc testing, cost-effectiveness

## Abstract

Background

Iron deficiency is the most common etiology of anemia among pregnant women. Many studies showed that anemia during pregnancy had been associated with adverse outcomes such as intrauterine growth retardation, preterm delivery, and maternal mortality. However, screening for those pregnant remains controversial.

Objectives

To find the prevalence of anemia among pregnant women and pregnancy outcomes. Also, to find the cost-effectiveness of running complete blood count (CBC) tests among them.

Methods

This is a retrospective record review done on pregnant women who delivered at King Abdulaziz University Hospital, Jeddah, Saudi Arabia, between January 1, 2018, and December 31, 2018. They were screened for eligibility, with the exclusion of those with hemoglobinopathies such as sickle cell anemia and thalassemia. Data were collected from their electronic medical records.

Results

A total of 5,120 pregnant women had delivered from January 1, 2017, to August 31, 2018, and 2,845 (55.6%) developed anemia during pregnancy. Out of 2,822, 2,301 were mild, 471 moderate, 50 severe, and 2,185 were normal. A total of 3,656 (71.4%) women were Saudis, and 1,464 (28.6%) were non-Saudis. The mean age was 29.85±6 years, and their first hemoglobin reading mean was 10.6±1.3g/dl. Out of 2,822, 546 developed undesired pregnancy outcomes. History of anemia, blood transfusion, intrauterine fetal demise, and stillbirth was significantly associated with abnormal hemoglobin levels (p<0.05). Complete blood count (CBC) testing for these pregnant women cost 422,990.92 US dollars.

Conclusion

Although the cut-off point of diagnosing anemia level during pregnancy isn’t fully understood, pregnant women with mild to moderate levels appeared to have lesser adverse pregnancy outcomes in comparison to women with severe level. Therefore, screening during prenatal visits or antenatal for anemia should be tailored to each pregnant based on her condition and the overall clinical judgment.

## Introduction

Anemia is a worldwide disorder affecting 1.62 billion people, and the prevalence of anemia in developing countries is much higher than in developed countries, 43%, and 9%, respectively. Decreased iron stores and anemia affect a large proportion of the population in developing and industrialized countries, especially groups at high risk as children, menstruating women, and especially pregnant women due to increase demand for iron during pregnancy period [1, 2].

Anemia in pregnant women and adolescent girls has serious health consequences [3]. It results both from nutrition-related causes and from inflammatory or infectious diseases, as well as from blood loss. Inadequate intake can result in iron-deficiency anemia, which is commonly seen during pregnancy, with an estimated prevalence of 30% to 50% for iron deficiency and 15% to 20% for iron deficiency anemia [4].

Low hemoglobin values during pregnancy and fetal health have been a source of continuing argument because around half of the world's women get anemia during pregnancy. Nevertheless, its influence on fetal development is a question of great importance in public health. There is evidence that severe anemia increases perinatal morbidity and mortality by causing intrauterine growth retardation and preterm delivery [5]. Moreover, it was estimated that anemia contributes to more than 115,000 maternal deaths and 591,000 prenatal deaths worldwide per year [2].

The lowest fall in hemoglobin level during pregnancy is seen during the 20^th ^week of gestation, and the concentration of hemoglobin remains constant up to 30 weeks and then rises slightly [6]. Thus, any estimation of hemoglobin concentration taken after the 20^th^ week of pregnancy will be reasonably representative of the fall induced by pregnancy. It is becoming clear that the best time to detect any perinatal risk associated with maternal anemia may be up until 20 weeks of gestation [7].

To the best of our knowledge, a lesser number of researches addressed this issue. Therefore, we wanted to study the prevalence of anemia among pregnant women attending obstetric clinics at King Abdulaziz University Hospital (KAUH). In addition, our primary objective was to evaluate the effect of low hemoglobin levels on antepartum and postpartum complications. The secondary objective was to clarify the cost-effectiveness of running multiple complete blood count (CBC) tests for those anemic pregnant and its financial burden on our hospital.

## Materials and methods

This study used data from electronic medical records of pregnant women, who had a hemoglobin level of less than 11 g/dL and had delivered between January 1, 2017, and August 31, 2018, at King Abdulaziz University Hospital (KAUH) Jeddah, Saudi Arabia. These records were obtained from the hospital’s Medical Records Department. The study’s ethical approval was obtained from the Institutional Review Board and the Research Ethics Committee of KAUH.

Pregnant women who were diagnosed with hemoglobinopathies (such as sickle cell anemia and thalassemia) were excluded. A total of 5,120 pregnant women were included for further analysis. According to the World Health Organization (WHO), hemoglobin levels of less than 11.0 g/dL is considered anemia in pregnancy [1]. The degrees of anemia were defined as mild (Hgb level of 9.0-10.9 g/dL), moderate (Hgb level of 7.0-8.9 g/dL), and severe (Hgb level of less than 7.0 g/dL) [3]. The subjects were subsequently divided into categories based on their degree of anemia. 

Maternal variables were the following: age, nationality, height, weight, gravidity, parity, previous abortions, type of pregnancy (singleton, multiple), known chronic illness, previous history of uterine surgery, and previous history of blood transfusion. Adverse antepartum variables included the following: antepartum hemorrhage (abruptio placenta, placenta previa, placenta accrete and low lying placenta), antiphospholipid syndrome (APS), gestational diabetes mellitus (GDM), impaired glucose tolerance test (GTT), induction of labor, maternal mortality, pregnancy-induced hypertension (PTH), preeclampsia, pre-premature rupture of membrane (PPROM), and premature rupture of membrane (PROM). Also, mode of delivery (spontaneous vaginal delivery [SVD] or Cesarean section [CS]), preterm labor (PTL), and type of CS (emergency or elective). 

Adverse neonatal outcomes included admission to neonatal intensive care unit, birth weight, gestational age, intrauterine fetal demise (IUFD), neonatal death, and stillbirth. Birth weight measures were taken as expected fetal weight (EFW). Gestational age was determined by the last menstruation period or craniocaudal length calculated by ultrasound during the first trimester of pregnancy. Moreover, we used the latest American College of Obstetrics and Gynecology (ACOG) guidelines in 2013 to define these variables [8].

For data management, first, it was reviewed in Microsoft Excel for Mac version 16. Data analysis was conducted using Statistical Package for Social Sciences (Version 26.0, IBM Inc., Armonk, USA) for Windows software. The univariate analysis included calculation of frequencies and percentages for categorical variables, and measures of central tendency were calculated for the continuous variables. Bivariate analysis such as the independent t-test and Pearson correlation tests were used for comparison of quantitative variables. Also, the chi-square test was used as a test of significance for the comparison of categorical variables. P-value ≤0.05 was chosen for statistical significance.

## Results

During the study period, a total of 5,120 women attended prenatal care. In the study population, age ranged from 12 to 56 years; mean was 29.85±6 years, gravidity ranged from one to 16; mean was 3.26±2.09, parity ranged from 0 to 10 with a mean of 1.89±1.75, and first hemoglobin reading ranged from 4.12 to 21.10; mean 10.6±1.34. Table 1 presents the socio-demographic of pregnant women. The majority of subjects were Saudis 3,656 (71.4%), and 1,464 (28.6%) were non-Saudis - also, most of the sample aged 20-34 years (73%). Multigravida (62.0%) was also more prevalent in the sample in comparison to primigravida (20.9%) and grand multigravida (13.7%). The prevalence of anemia in this study population was 2,822 (56.4%); among these, 46% had mild anemia, 9.4% had moderate anemia, and 1% had severe anemia, as shown in Table 1. Regarding the mode of delivery, most of our subjects had a spontaneous vaginal delivery with 3,336 (65.2%) cases.

**Table 1:** Socio-demographic characteristics and other obstetrical variables among the sample SVD - spontaneous vaginal delivery; CS - Cesarean section

Variables		
Age (years)	Mean (SD)	29.85 (6.08)
BMI	Mean (SD)	28.62 (5.77)
Nationality	Saudi n (%)	3,656 (71.4%)
	Non-Saudi n (%)	1,464 (28.6%)
Gravidity	Primigravida n (%)	54 (28.7%)
	Multigravida n (%)	134 (71.3%)
Hemoglobin	Mean (SD)	10.61 (1.35)
Age groups	<20	156 (3%)
	20-34	3,737 (73%)
	35-44	1,190 (23.2%)
	>45	37 (0.7%)
Hemoglobin levels (missing n=113)	Normal	2,185 (43.6%)
	Mild	2,301 (46.0%)
	Moderate	471 (9.4%)
	Severe	50 (1.0%)
Mode of delivery	SVD	3,336 (65.2%)
	Emergency CS	1,288 (25.2%)
	Elective CS	496 (9.7%)

When the patients were divided according to the hemoglobin levels into three degrees of anemia - mild anemia, moderate anemia, and severe anemia - the overall mild anemia was more prevalent among Saudis (76.3%) in comparison to non-Saudis (23.7%), while those who had severe anemia were 64%, and 36%, respectively. All degrees of anemia were commonly seen among Saudis (p=0.000). Furthermore, the relationship between maternal age and history of abortions to the degree of anemia was not statistically significant (p=0.265 and p=0.198, respectively), while in the case of gravidity, the difference it was statistically significant (p=0.001). Mild anemia was more common among pregnant women with multigravida G2-5 (64.5%), as shown in Table 2. A total of 662 cases from the sample had a history of previous uterine surgery. The data showed statistically significant differences in the degrees of anemia among those who had a blood transfusion and previous anemia diagnosis before pregnancy (p=0.000 and p=0.000, respectively).

**Table 2:** The prevalence of anemia and demographic variables

Variable		Hemoglobin levels				Total	P-value
		No anemia	Mild anemia	Moderate anemia	Severe anemia		
		n=2,185	n=2,301	n=471	n=50		
Nationality	Saudi	1,475 (67.5%)	1,756 (76.3%)	340 (72.2%)	32 (64.0%)	3,603	0.000
	Non-Saudi	710 (32.5%)	545 (23.7%)	131 (27.8%)	18 (36.0%)	1,404	
Age (years)	<20	57 (2.6%)	68 (3.0%)	21 (4.5%)	4 (8.0%)	150	0.265
	20-34	1,623 (74.3%)	1,675 (72.8%)	347 (73.7%)	35 (70.0%)	3,680	
	35-44	488 (22.3%)	546 (23.7%)	99 (21.0%)	11 (22.0%)	1,144	
	>45	17 (0.8%)	12 (0.5%)	4 (0.8%)	-	33	
Terminated as miscarriage (abortion)		2 (10.5%)	11 (57.9%)	6 (31.6%)	-	19	0.198
Gravidity	G1	79 (27.5%)	442 (21.5%)	66 (18.0%)	6 (17.6%)	593	0.001
	G2-G5	183 (63.8%)	1,323 (64.5%)	228 (62.1%)	23 (67.6%)	1,757	
	>G5	25 (8.7%)	287 (14.0%)	73 (19.9%)	5 (14.7%)	390	
Before pregnancy diagnosed anemia		2 (3.4%)	27 (46.6%)	20 (34.5%)	9 (15.5%)	58	0.000
Previous uterine surgery		75 (11.3%)	485 (73.3%)	91 (13.7%)	11 (1.7%)	662	0.463
Blood transfusion		9 (18.0%)	24 (48.0%)	12 (24.0%)	5 (10.0%)	50	0.000

Concerning maternal comorbidity, 313 out of 2,822 suffered from chronic diseases (see Table 3). Most of them had mild anemia with 234 cases compared to 48 and four cases in moderate and severe anemia, respectively. Hypothyroidism and bronchial asthma showed higher rates than other comorbidities with 145 and 64 cases, respectively. Furthermore, most of the cases with HTN and DM type 2 had mild anemia. On the other hand, iron deficiency anemia was statistically significant among other comorbidities (p=0.004) in mild and moderate anemia - 55% and 40%, respectively.

**Table 3:** The association between maternal comorbidity and degree of anemia HELLP - hemolysis, elevated liver enzymes, low platelet count; DM - diabetes mellitus; GERD - gastroesophageal reflux disease; HTN - hypertension

Maternal comorbidity	Hemoglobin levels				Total	P-value
	No anemia	Mild anemia	Moderate anemia	Severe anemia		
	n=2,185	n=2,301	n=471	n=50		
Antiphospholipid syndrome (HELLP syndrome)	0 (0%)	3 (100%)	0 (0%)	0 (0%)	3	0.630
Bronchial asthma	2 (3.7%)	40 (74.1%)	11 (20.4%)	1 (1.9%)	64	0.174
Iron deficiency anemia	0 (0%)	11 (55.0%)	8 (40.0%)	1 (5.0%)	20	0.004
DM type 1	1 (9.1%)	8 (72.7%)	2 (18.2%)	0 (0%)	11	0.926
DM type 2	4 (10.5%)	31 (81.6%)	2 (5.3%)	1 (2.6%)	38	0.367
Genetic disease	1 (7.7%)	10 (76.9%)	2 (15.4%)	0 (0%)	13	0.926
GERD	0 (0%)	2 (100%)	0 (0%)	0 (0%)	2	0.764
Hypothyroidism	17 (11.7%)	109 (75.2%)	17( 11.7%)	2 (1.4%)	145	0.889
HTN	4 (8.3%)	35 (72.9%)	9 (18.8%)	0 (0%)	48	0.501
Hyperthyroidism	0 (0%)	2 (66.7%)	1 (33.3%)	0 (0%)	3	0.717
Neurological disease (epilepsy, disc herniation)	1 (12.5%)	5 (62.5%)	2 (25.0%)	0 (0%)	8	0.791
Irritable bowel syndrome	0 (0%)	1 (50.0%)	1 (50.0%)	0 (0%)	2	0.611
Psychiatric illness	0 (0%)	2 (100%)	0 (0%)	0 (0%)	2	0.764
Hyperlipidemia	0 (0%)	1 (50.0%)	1 (50.0%)	0 (0%)	2	0.611

As shown in Table 4, a total of 1,175 (41.3%) developed undesired pregnancy outcomes with the majority of gestational diabetes mellitus (GDM) among 250 study subjects. Moreover, premature rupture of membrane (PROM) ranked second undesired outcome, in which most of the cases had mild anemia (72.3%). Mild and severe preeclampsia were commonly seen among pregnant women with mild anemia levels with 10 and 7 cases, respectively. In the case of adverse neonatal outcome, intrauterine fetal death (IUFD) and neonatal death were commonly seen among the mild anemia group with 13 cases in each. The relationship between hemoglobin levels and IUFD was statistically significant (p=0.026). Preterm birth was seen in 39 out of 61 (63.9%) cases, mostly in the mild anemia group. Neonatal intensive care unit (NICU) admission was frequent in the mild anemia group with 15 cases (57.7%).

**Table 4:** Pregnancy outcomes among the sample GDM - gestational diabetes mellitus; GTT - glucose tolerance test; HTN - hypertension; NICU - neonatal intensive care unit

Pregnancy Outcomes	Hemoglobin levels				Total	P-value
	No anemia	Mild anemia	Moderate anemia	Severe anemia		
	n=2,185	n=2,301	n=471	n=50		
Abruptio placenta	0 (0%)	3 (100%)	0 (0%)	0 (0%)	3	0.630
GDM	27 (10.8%)	182 (72.8%)	38 (15.2%)	3 (1.2%)	250	0.843
Antepartum hemorrhage	0 (0%)	0 (0%)	1 (100%)	0 (0%)	1	0.260
Impaired GTT	0 (0%)	4 (66.7%)	2 (33.3%)	0 (0%)	6	0.440
Placenta previa	0 (0%)	3 (100%)	0 (0%)	0 (0%)	3	0.630
Premature rupture of membrane	8 (8.5%)	68 (72.3%)	15 (16.0%)	3 (3.2%)	94	0.402
Severe preeclampsia	2 (20.0%)	7 (70.0%)	1 (10.0%)	0 (0%)	10	0.781
Mild preeclampsia	0 (0%)	10 (83.3%)	2 (16.7%)	0 (0%)	12	0.393
Placenta accrete (increta, percreta)	0 (0%)	2 (100%)	0 (0%)	0 (0%)	2	0.764
Pre-premature rupture of membrane	3 (25.0%)	9 (75.0%)	0 (0%)	0 (0%)	12	0.150
Pregnancy induced HTN	0 (0%)	8 (80.0%)	2 (20.0%)	0 (0%)	10	0.450
Low lying placenta	0 (0%)	1 (100%)	0 (0%)	0 (0%)	1	0.902
Intrauterine fetal death	2 (8.3%)	13 (54.2%)	9 (37.5%)	0 (0%)	24	0.026
Stillbirth	3 (10.3%)	16 (55.2%)	9 (31.0%)	1 (3.4%)	29	0.061
Neonatal death	2 (9.5%)	13 (61.9%)	6 (28.6%)	0 (0%)	21	0.287
NICU admission	5 (19.2%)	15 (57.7%)	5 (19.2%)	1 (3.8%)	26	0.241
Preterm birth	6 (9.8%)	39 (63.9%)	14 (23.0%)	2 (3.3%)	61	0.113

Finally, regarding the cost, from 2017 to 2018, a total of 7,942 tests were done - 5,120 tests for the first time antenatal visit and 2,822 second tests for anemic patients. In our hospital, a single CBC testing with differentiation costs 53.26$ US dollars. The total estimated cost of the CBC tests with differentiation was 422,990.92$ US dollars.

## Discussion

This study assessed the prevalence of anemia among pregnant women attending KAUH and evaluated the effects of low Hb-level on antepartum and postpartum outcomes. Moreover, it addressed the cost-effectiveness of running multiple complete blood count (CBC) tests for the anemic pregnant women and their costs. Anemia is considered to be one of the risk factors for adverse maternal and neonatal outcomes among pregnant women. Therefore, effective measures such as early intervention with iron supplements had helped to decrease undesired outcomes. Interestingly, none had investigated the cost-effectiveness of running multiple CBC testing among high-risk group populations, such as pregnant women [9]. Nevertheless, according to a recent study, half of those who were anemic in the first antenatal clinic had a resolution while the rest were still anemic [10]. 

Enrera et al. reported that the prevalence of anemia in Saudi Arabia among pregnant women is 58% compared to our result, which was 55.6% [11]. A recent study published by the WHO showed a reduction in the prevalence of anemia globally among pregnant women from 43% to 38% over the last decade [12]. The variance between these findings could be related to reduced oral intake of iron supplements or inadequate consumption of red meat products. For example, Al-Quaiz et al. showed that the rate of consumed red meat in Riyadh was less than two times per week [13]. Also, a multicenter study in the US reported that a low intake of red meat among healthy women was picked up either as an incident finding or persistent anemia [13, 14]. In developed countries, socioeconomic status can play a significant role [15].

According to the literature, maternal age, and the degree of anemia among pregnant women is considered to have a significant relationship [16]. However, our result showed no statistical significance between maternal age and the severity level of anemia. Moreover, our study showed a significant relationship between anemia and those who were multigravida, supporting the result of a study done in Trinidad and Tobago where it was found that multigravida was more susceptible to anemia than primigravida [17]. This variation in studies could be due to the differences between sociodemographic characteristics among study participants. 

Regarding pregnancy-related complications, we found that the majority of our sample did not develop any undesired maternal or neonatal outcomes. However, the study showed that 21.3% of our sample developed GDM with no significant relationship with anemia prevalence during pregnancy. This result was matched to an African cross-sectional study that found no significant relationship with GDM incidence and anemia of pregnancy [18]. This could be due to multiple GDM related factors such as the previous diagnosis of GDM or increased susceptibility in obese patients and other factors beyond our study focus.

Furthermore, another significant relationship was found between anemic pregnant women and an emergency Cesarean section, and it was more prominent in the mild anemia group, followed by the groups with moderate and severe levels, respectively. Similarly, the Cesarean section was a significant complication among patients with low Hb levels during their first trimester, according to a big Turkish study conducted in 2016 and another African study published in 2015 [18, 19]. However, a study conducted in Saudi Arabia had found no significant relationship between pregnant women with anemia and an increase in the number of CS [20]. There was an apparent difference between our study and the Turkish study compared to the Saudi article in terms of representativeness of the data, which may partially explain the controversy regarding the link between performing CS and anemia in pregnancy. A recent Indonesian study examined a significant association between anemic pregnant women and PROM while we found no significant relationship between them [21].

This study showed a statistical significance between anemic pregnant women and intrauterine fetal death. Another study done in Pakistan documented that anemic pregnant patients are 2.5 times more likely to have intrauterine fetal death than non-anemic, however, this result was not statistically significant [5]. Our results were similar to a Tanzanian study carried out in 2018, which found that premature birth, as well as low birth weight, were not significantly associated with anemia among pregnant women [22]. Nevertheless, a local study conducted in Makkah demonstrated a statistical significance between low birth weight and anemic pregnant women [20]. Furthermore, a recent article found that low birth weight and premature birth were significantly linked with anemic pregnant women in the first trimester, including a recent systematic review and meta-analysis [22, 23]. Overall, we had low percentages of pregnancy-related insults compared to other studies, and this, according to Stephen et al., could justify the lack of significance in our study [22].

According to the most recent update on the US Preventive Services Task Force (USPSTF) recommendations, there is insufficient evidence regarding the benefits of screening for iron deficiency anemia (IDA) in asymptomatic pregnant women [24]. Other guidelines, such as the American College (ACOG, 2008) and Centers for Disease Control and Prevention (CDC, 1998), recommend screening for asymptomatic IDA; however, do not provide information regarding the appropriate time for screening and whether to screen at several intervals during the pregnancy [25, 26]. In contrast, the Institute of Medicine (IOM, 1993) guidelines recommend screening at each trimester [27]. A study showed a difference in screening practices among general obstetrics-gynecology practitioners where results were divided between screening at initial visits only and screening at every trimester [28]. A routine CBC test to measure serum hemoglobin or hematocrit levels is frequently used for assessment. Routine screening for IDA may have its benefits and consequences. It may lead to earlier identification and management of IDA, and the prevention of adverse outcomes, such as preterm delivery, low birth weight, and impairment of fetal development, where iron is required, particularly for brain development [29]. Nevertheless, a study highlighted the possible consequences of routine screening, such as unnecessary excess costs in low-risk patients [30].

To our knowledge, a cost-effectiveness analysis of routine IDA screening among pregnant women was not done before. According to our study, we estimated that the total cost of CBC tests with differentiation done from 2017 to 2018 was 422,990.92$ US dollars, which constitutes 7,942 tests for a total of 5,120 patients. Our results showed no remarkable association between lower hemoglobin levels and adverse maternal nor neonatal outcomes, as the majority of cases did not develop any, which further supports the USPSTF's latest statement regarding the insufficient evidence to proceed with screening for asymptomatic pregnant women. 

Concerning the limitation, this is a retrospective, single-center study and we cannot generalize our findings among the western region population or to the other regions of Saudi Arabia. For that reason, a more comprehensive study is needed to assess the overall impact of anemia among all pregnant women not only in the western region but in all regions of Saudi Arabia.

## Conclusions

As our study showed, pregnant women with mild anemia, which were the majority in our sample, did not develop adverse maternal or neonatal outcomes compared to the non-anemic group. Thus, doing multiple CBC tests during antenatal without a valid reason will not help in changing pregnancy outcomes. In addition, implementing a broad plan targeting pregnant women’s diet with a proper health education prior to their pregnancy will help them minimize their risk of developing anemia, which is a known risk factor for adverse maternal and neonatal outcomes.
